# Major HIV resistance mutations in untreated Romanian patients

**Published:** 2011-05-25

**Authors:** A Temereanca, L Ene, D Duiculescu, S Ruta

**Affiliations:** * ‘Carol Davila’ University of Medicine and Pharmacy, Faculty of Dentistry, BucharestRomania; ** ‘Stefan S. Nicolau’ Virology Institute, BucharestRomania; *** ‘Dr. Victor Babes’ Hospital for Infectious and Tropical Diseases, BucharestRomania

**Keywords:** HIV, subtype F, naive patients, transmitted drug resistance

## Abstract

Drug resistance mutations are frequently detected in antiretroviral–naive HIV positive patients, however the data on transmitted resistance in non-B subtypes are limited. As HIV1 subtype F is prevalent in Romania, our goal is to analyze resistance mutations in the pol gene of HIV–1 isolates from drug–naive Romanian patients. HIV–1 pol gene from 12 untreated patients, newly diagnosed (n = 6) and chronically infected (n=6), with detectable HIV RNA viral load was genotyped and the viral subtype was determined by using the Stanford database algorithm. 8/12 strains belonged to the F subtype, 1/12 to the G subtype, and the rest of the studied strains appeared to be K/F, A/F and J/F inter–subtype recombinant forms. The prevalence of HIV–1 strains with at least one major drug resistance mutation in the studied group was unexpectedly high. Major mutations associated with NRTI, NNRTI and PI resistance were detected in 6/12 patients, 2/12 patients and 3/12 patients, respectively; in addition all viral strains had minor mutations in the protease gene. Newly diagnosed patients harbored resistant variants more often than chronically infected ones (4/6 vs. 2/6) did. These data support the use of genotypic resistance testing in treatment–naive HIV positive patients, in order to guide the selection of the first line of antiretrovirals, due to the fact that persons with transmitted drug resistance have a higher risk for both virologic failure and development of resistance at treatment initiation.

HIV–Human immunodeficiency virus; TDR–transmitted drug resistance; HAART–highly active antiretroviral therapy ; SDRM–surveillance list of drug resistance mutations ; NRTIs–nucleos(t)idic reverse–transcriptase inhibitors; NNRTIs– non–nucleosidic reverse transcriptase inhibitors; PIs–protease inhibitors; TAMs–thy–midine analogue mutations; 3TC –lamivudine ; FTC–emtricitabine ; ddI –didanosine ; ABC–abacavir ; ZDV–zidovudine ; d4T–stavudine ; TDF –tenofovir

## Introduction

The transmission of drug–resistant HIV–1 has important implications for the successful management of antiretroviral therapy among infected individuals, restricting drug options and increasing the risk of suboptimal treatment outcomes. Persons with transmitted drug resistance (TDR) begin antiretroviral therapy with a lower genetic barrier to resistance, a higher risk of virologic failure, and a higher risk of developing resistance even to those drugs in their regimen that were originally fully active [1–3]. For these reasons, the new guidelines recommend to perform the genotypic resistance testing in all drug naive patients, before beginning a first line antiretroviral regimen [4]. 

To date, there is a growing literature about the rate of transmission of HIV–1 drug–resistant viruses. In the United States and in Europe, where there is a wide access to highly active antiretroviral therapy (HAART), the prevalence of HIV–1 drug–resistant strains ranges between 3.3Z% and 14.0% in recently infected patients and between 6.1% and 12.5% in chronically infected ones [5–10]. To accurately compare transmitted drug resistance rates across geographic regions and times, the World Health Organization has recommended the adoption of a consensus genotypic definition of transmitted HIV–1 drug resistance. Currently, a surveillance list of drug resistance mutations (SDRM) is constantly updated. The last updated SDRM list from 2009 has 93 mutations including 34 NRTI–resistance mutations at 15 RT positions, 19 NNRTI–resistance mutations at 10 RT positions, and 40 PI–resistance mutations at 18 protease positions [11].

The detection of transmitted HIV–1 drug resistance is dependent on various factors. In the absence of therapy, resistance mutations may decline over time and become undetectable by current assays, but may persist archived and cause treatment failure when ART is started. The identification of TDR is further complicated by the diversity of HIV across different types (HIV–1 and HIV–2), groups (main [M], outlier [O] and new [N]), subtypes and recombinant forms [12, 13–16] that are prevalent worldwide [17]. Group M accounts for over 99% of globally reported HIV/AIDS infections, and, is further classified into nine pure subtypes (A, B, C, D, F, G, H, J and K) and many circulating and other recombinant forms. HIV–1 nonsubtype B variants account for the majority of HIV infections worldwide, but are the least studied; most existing knowledge of HIV–1 pathogenesis and responsiveness to antiretroviral therapy is based on work carried out with subtype B viruses (most common in North America, Europe, and Australia). 

Previous studies showed that subtype F largely dominates the epidemiology of HIV–1 infection in Romania [18], where a major pediatric HIV epidemic with several thousand cases infected mainly through blood transfusions and injections with improperly sterilized equipment was reported at the end of '80s. According to the National Report of the HIV/AIDS Monitoring and Evaluation Department in Romania (http://data.unaids.org), at the end of 2009, a cumulative total of 16,162 cases of HIV and AIDS infection had been recorded, with 10,041 persons living with HIV/AIDS. The largest age group of people living with HIV/AIDS consists of young people (17–21 years) over 6,000, which are in fact long time survivors, infected between 1987 and 1992. The incidence of HIV/AIDS newly diagnosed cases decreased in Romania starting from 2004. The number of newly diagnosed patients in 2008 and 2009 was of 436 and 428, respectively. Almost 50% of the newly cases of HIV/AIDS discovered in 2009 were among young persons aged 15 to 29. 

Considering the fact that data on non–B subtypes is limited, and subtype F is prevalent in Romania, our goal is to analyze resistance mutations in the pol gene of HIV–1 isolates from antiretroviral–naive Romanian patients. 

## Patients and methods

This is a pilot study of 12 antiretroviral therapy naives HIV–1 infected patients, with clinically followed–up in ‘Dr. Victor Babes’ Hospital in Bucharest. All patients had detectable HIV viral load, as determined by plasma HIV–1 RNA quantification (COBAS AMPLICOR HIV–1 MONITOR TEST version 1.5; lower detection limit, 400 copies/ mL ARN HIV–1; linear range between 400 (log_10_=2.6) to 750,000 (log_10_=5.87) copies/mL ARN HIV–1). Drug resistance genotyping was performed using the TruGene HIV–1 Genotyping Assay (SIEMENS). This technique consists of an RT reaction performed on HIV RNA extracted from plasma, followed by PCR amplification of a 1,318–bp fragment of the pol gene and CLIP sequencing of the PCR amplicons using HIV–1 specific primers. Separation of the CLIP sequencing reactions by electrophoresis on a polyacrylamide gel, and detection by laser–induced fluorescence is followed by analysis using the OpenGene DNA System Software, with a HIV–1 Resistance Report issued for each sample. For subtyping purposes and drug resistance interpretation, all sequences were submitted to the Stanford University HIV database. Mutations associated with transmitted drug resistance (TDR) were identified using the WHO 2009 list of mutations for surveillance of TDR HIV strains http://hivdb.stanford.edu/pages/WHOResistanceList.html.

## Results

**Study population**. All 12 study patients were naive for therapy at the time of HIV resistance testing: 6 were newly diagnosed cases of HIV infection, and 6 were known to be chronically infected (for more than 1 year). There were 6 males and 6 females, with a median age of 30,5 years (range from 7 to 39 years). In the group of newly diagnosed patients (mean age 33.8 years) the transmission route was heterosexual contact, while chronically infected patients (mean age of 21 years) were long–term survivors, with HIV parenterally acquired during childhood. There were no significant differences in the median CD4+ cell count in the two subgroups of patients (232 cell/mm^3^ for newly diagnosed patients vs 303 cell/mm^3^ for chronically infected ones). The median HIV RNA viral load was higher in newly diagnosed individuals (235 400 HIV RNA copies/mL) than in persons with chronic infection (126 650 HIV RNA copies/mL). HIV–1 subtype analysis indicated that 8 out of the 12 strains belonged to the F subtype, one to the G subtype, while the rest appeared to be K/F; A/F or J/F inter–subtype recombinant forms.

**Figure 1:**
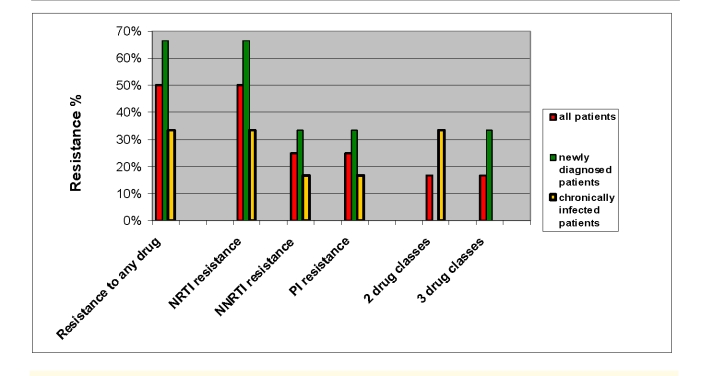
Prevalence of primary resistance. Half of the studied patients were harboring primary HIV–1 drug resistance mutations, mainly associated with NRTI resistance. Newly diagnosed patients carried resistant variants more often than did chronically infected ones (4/6; 66.6% vs. 2/6; 33.3%). Resistance to 2 drug classes was present in 2/12 (16.6%) patients, and resistance to all three drug classes was observed in other 2 cases.

**Prevalence of drug resistance mutations**. Among the 12 drug–naive patients included in the study, 6 carried HIV strains with at least one resistance–related mutation (Figure 1).

Newly diagnosed patients harbored resistant variants more often than the chronically infected ones (4/6; 66.6% vs. 2/6; 33.3%) did. Resistance to 2 drug classes was present in 2/12 (16.6%) patients, and resistance to all three drug classes was observed in other 2 cases. 

Mutations associated with resistance to nucleoside reverse transcriptase inhibitors (NRTIs) were detected most frequently (in 6 out of 12 patients), followed by non-nucleoside reverse transcriptase inhibitor (NNRTI) resistance mutations (in 3 out of 12 patients) and protease inhibitor (PI) resistance mutations (in 3 out of 12 patients). 

Major mutations associated with NRTI resistance were observed in 6 out of the 12 studied patients. Mutations known to be selected by thymidine analogues, termed TAMs were the most predominant (5/12; 41,6%), TAMs 2 being more frequently detected (3/12; 25%): K70R and K219Q (each in 2 cases), D67N and T215F (each in one case)–all associated with resistance to AZT, d4T, 3TC. The only TAM1 observed was M41L, found in 2 cases. In vitro, this mutation causes high–level resistance to lamivudine (3TC) and emtricitabine (FTC), low–level resistance to didanosine (ddI) and abacavir, (ABC) and increased susceptibility to zidovudine (ZDV), stavudine (d4T), and tenofovir (TDF). The revertant mutation at codon 215 (T215N), associated with an increased risk of virologic failure was identified in one drug–naive individual. M184V, the 3TC–selected mutation, was found in only one patient together with all the above mentioned TAMs.  

Major mutations K103N and Y181C associated with primary NNRTI resistance were observed each in one of the newly diagnosed patients.

Regarding PI resistance, major mutations were observed in 3 out of the 12  drug–naive patients with V82A/S/T present in all cases, either alone or accompanied by I84L in one case and M45I, I47V, I54V in the other one. 

All the studied HIV–1 strains had minor mutations in the protease gene. M36I, was the most common accessory protease inhibitor resistance mutation, found in all studied patients, and followed by E35D (in 10 cases),K20R and I15V (in 9 cases). M36I is one of the polymorphic substitutions in subtype F and other non–B HIV proteases that may lead to early development of drug resistance in patients infected with non–B subtypes. The L89M polymorphism, the most prevalent signature among treatment–naive non–subtype B isolates, was found in 9/12 patients. Mutations L63T (present in 7 cases) and L10V (present in 3 cases) have been suggested as predictors of virological failure in patients who had been treated with multiple antiretroviral drugs.

## Discussion

We have found a strikingly high HIV–1 TDR mutation rate in our small group of patients. Half of the studied patients were harboring primary HIV–1 drug resistance mutations, mainly associated with NRTI resistance. This is the first report on the presence of major mutations in treatment–naive HIV–positive Romanian patients. A previous study conducted in ‘Matei Bals’ Institute of Infectious Diseases on a sample of 29 drug–naive HIV–positive patients revealed no major mutations associated with resistance to NRTIs, NNRTIs, and PIs [19].  

The baseline resistance was more common in newly diagnosed individuals than in chronically infected patients (66.6% vs 33.3%). The lower prevalence of resistance in chronic infection is most likely due to a lower exposure to drug‐resistant virus in the past. In addition, the lower prevalence can be explained by reversion from resistant variants to sensitive wild‐type viruses over time [20]. Interestingly, drug–resistance mutations could still be detected in the plasma of quite a few chronically infected patients, which indicates that complete reversion does not always occur. Indeed, many studies have indicated that drug-resistant viruses can persist for a couple of years in the plasma of treatment‐naive HIV‐infected patients [21, 22].  

The persons who have been infected for more than 1 year are considered to be chronically infected. The duration of HIV infection in newly diagnosed individuals is usually unknown and can be highly variable. To differentiate recent from chronic HIV–1 infections among newly diagnosed patients it is necessary to perform the BED IgG–Capture enzyme immunoassay (BED–CEIA). The recent infection is confirmed by this method, which is based on the principle that characteristics of the initial HIV antibody response in recent infections differs from those of established or long–term infections either by antibody titer, proportion, specificity, isotype or avidity.

We also found that half of our subjects harbour HIV–1 strains with at least one major drug resistance mutation. 

The most frequent substitutions of amino acids in reverstranscriptase gene belong to the TAM category, a fact that may compromise the efficacy of most drugs from this class. It is interesting to note that although both TAM–1 and TAM–2 pathway mutations were encountered, the TAM–2 pathway was the better represented, in contrast with the available data obtained from subtype B strains [23]. Type I TAM cause higher levels of phenotypic and clinical resistance to the thymidine analogs and cross–resistance to abacavir, (ABC), didanosine (ddI), and tenofovir (TDF), than do the type II TAMs [24].

Mutation M184V was found in only one HIV–1 strain, subtype F, together with D67N, T69D, K70R, L74I, T215F and K219Q. As this mutation tends to be rapidly archived, its presence together with the other mentioned TDR suggests a recent infection with a resistant HIV–1 strain. The phenotypic and clinical significance of M184V is influenced by the presence or absence of other NRTI resistance mutations. Four TAMs in combination with M184V cause high–level resistance to both ABC and ddI [25–29]. The presence of the T215 revertant suggests a previous infection with a HIV–1 strains containing T215Y/F [30, 31], and has been associated with an increased risk of virologic failure in patients receiving a first line regimen with thymidine analogue [32]. Mutations K103N and Y181C, presented in 2 newly diagnosed patients, confer cross–resistance to all NNRTIs. Major mutations associated with PI resistance were observed at positions 46, 47, 54, 82 and 84, conferring resistance to the most important PIs used in antiretroviral treatment

All the studied HIV–1 strains carried many minor mutations with high frequencies in the protease gene. These mutations are often found at polymorphic positions and may have limited effect on susceptibility to antiretroviral drugs. However, these mutations should be considered when assessing the possible impact on the therapy response as they have been reported to be associated with high viral fitness in strains with major mutations [33,34,35,39]. One consequence of preexisting accessory mutations might be the faster emergence of viruses resistant to PIs. 

We observed the presence of TDR in 2 patients who had risk factors in acquiring the infection in early childhood, and therefore we considered them to be parenterally–infected. As an infection with a TDR viral strain at the beginning of HIV epidemic is doubtful, we may take into consideration the possibility of a superinfection with a resistant HIV–1 recently acquired, probably by a sexual route. 

This high rate of TDR in patients infected with F subtype is conflicting with previous European reports regarding TDR, where non–B subtypes were harboring less TDR mutations [40, 41]. The explanation can be related to the fact that HIV epidemic in Romania has been evolving over 2 decades and many patients have been exposed to antiretroviral therapy. Some of them, failing ARV regimens, are prone to transmit HIV–1 strains with TDR mutations. Also the group of children infected in the early 1990 became sexually active and from an epidemiological point of view represent a group with high risk to transmit HIV-resistant strains [42]. 

Regarding the subtype distribution in our study, even if F subtype remains prevalent, we observed an increase of HIV–1 infections which involved other subtypes and inter–subtype recombinant forms, aspect that has to be further confirmed by detailed genotyping studies. We could not find a difference between the rates of TDR in patients infected with F strain, compared to those infected with other strains. 

From the point of view of TDR subtype, diversity raises some questions because of polymorphism mutations (drug resistance mutations that occur commonly in the absence of drug selective pressure). For C clade, baseline polymorphism at codon 106 has facilitated the development of a novel V106M mutation, conferring efavirenz resistance [43]. Protease inhibitor mutations L89I/V have been reported in C, F and G subtypes [44]. A review on non–B subtype performed by Cajas and collaborators showed that polymorphisms that were common in non–B subtypes and that may contribute to resistance, tended to persist or become more frequent after drug exposure. The authors outlined that some, but not all are recognized as minor resistance mutations in B subtypes, and that these observed differences in resistance pathways may impact cross–resistance and the selection of second–line regimens with protease inhibitors. Attention to newer drug combinations, as well as baseline genotyping of non–B isolates, in well–designed longitudinal studies with long duration of follow up are needed.

The limitation of our study is related to the small study group. Further studies are needed in order to evaluate if this high rate reflect the present situation in Romania, where many patients have been exposed to antiretroviral treatment for over a decade, and if they are failing their current regimens and might transmit HIV–antiretroviral resistant strains.  

## Conclusion

The prevalence of HIV–1 strains with at least one major drug resistance mutation in the studied group was very high, major mutations being more frequently identified among newly diagnosed patients compared to chronically infected patients. These data supports the use of genotypic resistance testing in treatment–naive HIV positive patients, especially in newly diagnosed ones, in order to guide the selection of the first line of antiretroviral treatment.

Acknowledgements: The authors thank Dr. Codruta Vagu and Petruta Mihaila for technical support.   

Sources of Funding : This work was supported by Grant No. 5 P30 AI036211–15 rev awarded by the National Institutes of Health (NIH), Public Health Service (PHS) to Baylor College of Medicine, subcontract  PO 5600167489 between Baylor College of Medicine, Houston, Texas, USA and ‘Stefan S. Nicolau’ Institute of Virology, Bucharest, Romania.
